# Infant Cancer in Taiwan: Incidence and Trends (1995-2009)

**DOI:** 10.1371/journal.pone.0130444

**Published:** 2015-06-25

**Authors:** Giun-Yi Hung, Jiun-Lin Horng, Hsiu-Ju Yen, Chih-Ying Lee

**Affiliations:** 1 Division of Pediatric Hematology and Oncology, Department of Pediatrics, Taipei Veterans General Hospital, Taipei, Taiwan; 2 Department of Pediatrics, National Yang-Ming University School of Medicine, Taipei, Taiwan; 3 Department of Anatomy and Cell Biology, School of Medicine, College of Medicine, Taipei Medical University, Taipei, Taiwan; National Health Research Institutes, TAIWAN

## Abstract

**Background:**

Current information about cancer incidence patterns among infants in East Asia is rare. The objective of this study was to report the first population-based cancer surveillance of infants in Taiwan.

**Methods:**

Cancer frequencies and incidence rates among subjects aged <1 year for the period 1995-2009 were obtained from the Taiwan Cancer Registry. Types of cancers were grouped according to the International Classification of Childhood Cancer. Rates and trends were analyzed by sex and disease groups and further compared with that of other countries.

**Results:**

A total of 900 infants were diagnosed with cancers, giving an incidence rate of 250.7 per million person-years from 1995 to 2009. The male-to-female incidence rate ratio was 1.22. Overall, leukemias (56.3 per million) were the most common cancer, followed by germ cell neoplasms (43.2) and neuroblastomas (41.8). The incidence increased by 2.5% annually during the 15-year study period and was predominantly contributed by male infants (3.5%). Compared with other countries, the rate of hepatoblastoma in Taiwan was second to that from Beijing (China) and 2 to 5 times greater compared with the US, France, the North of England and Osaka (Japan). The rates of germ cell neoplasms were 2 to 4 times greater in Taiwan.

**Conclusions:**

The current data suggests that cancer incidence rate among male infants was rising in Taiwan. The factors associated with higher rates of hepatoblastoma and germ cell neoplasms warrant further investigation on similar ethnic groups of different areas to elucidate the potential environmental impacts while controlling for race.

## Introduction

The age of peak cancer incidence among children occurs during the first year of life. There are substantial differences for cancers among infants compared to older children, in terms of incidence, anatomical site, histological features and clinical behavior [1,2]. Aberrant genetic processes could occur in embryos and the process of carcinogenesis is compressed into a relatively brief period. Because the potential exposure period will only include the prenatal period and perhaps a few months postnatal, it may be easier to study environmental and constitutional genetic factors and their interactions on infant cancers [3]. Research into the causes of these cancers can proceed in the laboratory, where a better understanding of the genetic and cellular process involved in malignant transformation can provide clues to possible etiological factors, or can be based on epidemiological observation [1,4]. However, the rarity and diversity of infant cancers make epidemiological surveillance difficult to perform.

In 2009, infants aged less than 1 year accounted for only 0.8% of the total population in Taiwan [5]. The incidence of cancer is low among these infants, comprising 0.05% of all cancer cases [6]. Unlike adult cancers that are commonly tabulated by primary sites, cancers in children are grouped more appropriate according to histology and primary site based on the International Childhood Cancer Classification (ICCC) [7]. Among all studies published in English, only 3 studies from East Asia reported childhood cancer incidence rates that included the rates of infants according to ICCC, based on population-based cancer registries [8–10]. These studies mainly described childhood cancer, lacking clear details specific for infant cancer. By examining the data extracted from population-based Taiwan Cancer Registry (TCR) database over a 15-year period, this study aimed to investigate the cancer incidence and temporal trends specifically for infants, with respect to further research into causes of these cancers as well as public health importance.

## Materials and Methods

### Data collection

Incidence and census data in this study were obtained from the TCR, which is organized and funded by the Health Promotion Administration, Ministry of Health and Welfare, Taiwan. The registration of population-based TCR for all cancers was initiated in 1979 [6,11]. After the enactment of the Cancer Control Act in May 2003, hospitals with a capacity of ≥ 50 beds that provided hospitalized cancer care were mandated to submit cancer data to the central cancer registry, which enhanced the completeness of registration, case ascertainment and the quality of cancer data collection. The number of hospitals participated increased from 167 in 1995 to 213 in 2009. In addition, the current health care system in Taiwan, known as National Health Insurance (NHI), a compulsory social insurance plan which centralizes the disbursement of health-care funds, was launched in 1995 [12]. The system promises equal access to health care for all citizens and the population coverage of NHI had reached 99% by the end of 2004. In terms of data quality of the TCR according to the quality indicators defined by the International Agency for Research on Cancer (IARC), the percentage of microscopically-verified cases (MV%) increased from 81.26% in 1995 to 90.75% in 2009 for all cancers combined; although it varies according to the type of cancer [11,13]. The percentage of death certificate only cases (DCO%) is another indicator of data validity; it fell from 19.63% in 1995 to 1.15% in 2009. These indicators described above reveal the high quality of the TCR and its remarkable improvement over time.

The incidence data for all cancers among infants (<1 year old) diagnosed from 1995 to 2009 were analyzed. Data of other pediatric ages (1–14 years) were also extracted for comparison. Only cases diagnosed with malignant tumors were included in the data. Diagnoses were classified into 12 main groups and 47 subgroups according to the ICCC version 3 (ICCC-3) [7]. The cases presenting tumors that were not classified by the ICCC-3 or cancers in situ were excluded from the final analysis in order to compare the incidence with other countries. We abbreviated 7 of the 12 major ICCC-3 groups as follows: (1) leukemias: leukemias, myeloproliferative and myelodysplastic diseases; (2) lymphomas: lymphomas and reticuloendothelial neoplasms; (3) central nervous system (CNS) neoplasms: CNS and miscellaneous intracranial and intraspinal neoplasms; (4) neuroblastomas: neuroblastoma and ganglioneuroblastoma; (5) soft tissue sarcomas: soft tissue and other extraosseous sarcomas; (6) germ cell neoplasms: germ cell tumors (GCTs), trophoblastic tumors, and neoplasms of the gonads; and (7) other epithelial neoplasms: other malignant epithelial neoplasms and malignant melanomas.

### Analyses

Rates were expressed per million person-years according to sex and were presented in accordance with the ICCC-3 into the main groups and subgroups described above. Rates were calculated according to the methods published previously [14]. We identified changes in trend during the study period using Joinpoint regression models and permutation tests (Joinpoint Regression Program, Version 4.0.4) to identify significant changes [15,16], in which up to 3 joinpoints were produced to express the annual percent changes (APC). This software takes trend data and fits the simplest joinpoint model that the data allow. The program starts with the minimum number of joinpoints and tests whether more joinpoints are statistically significant and must be added to the model. The grid search method described by Lerman (1980) was used to fit the segmented regression function, the *P* value of each permutation test was estimated using Monte-Carlo methods, and the overall asymptotic significance level was maintained through a Bonferroni adjustment [15]. The APC was considered significant if the 95% confidence interval (CI) did not include zero. To compare our data with the cancer incidence data for infants from other countries, we compiled world rates by searching the databases available from the internet (Medline, National Center for Biotechnology information, PubMed).

## Results

**Fig 1:**
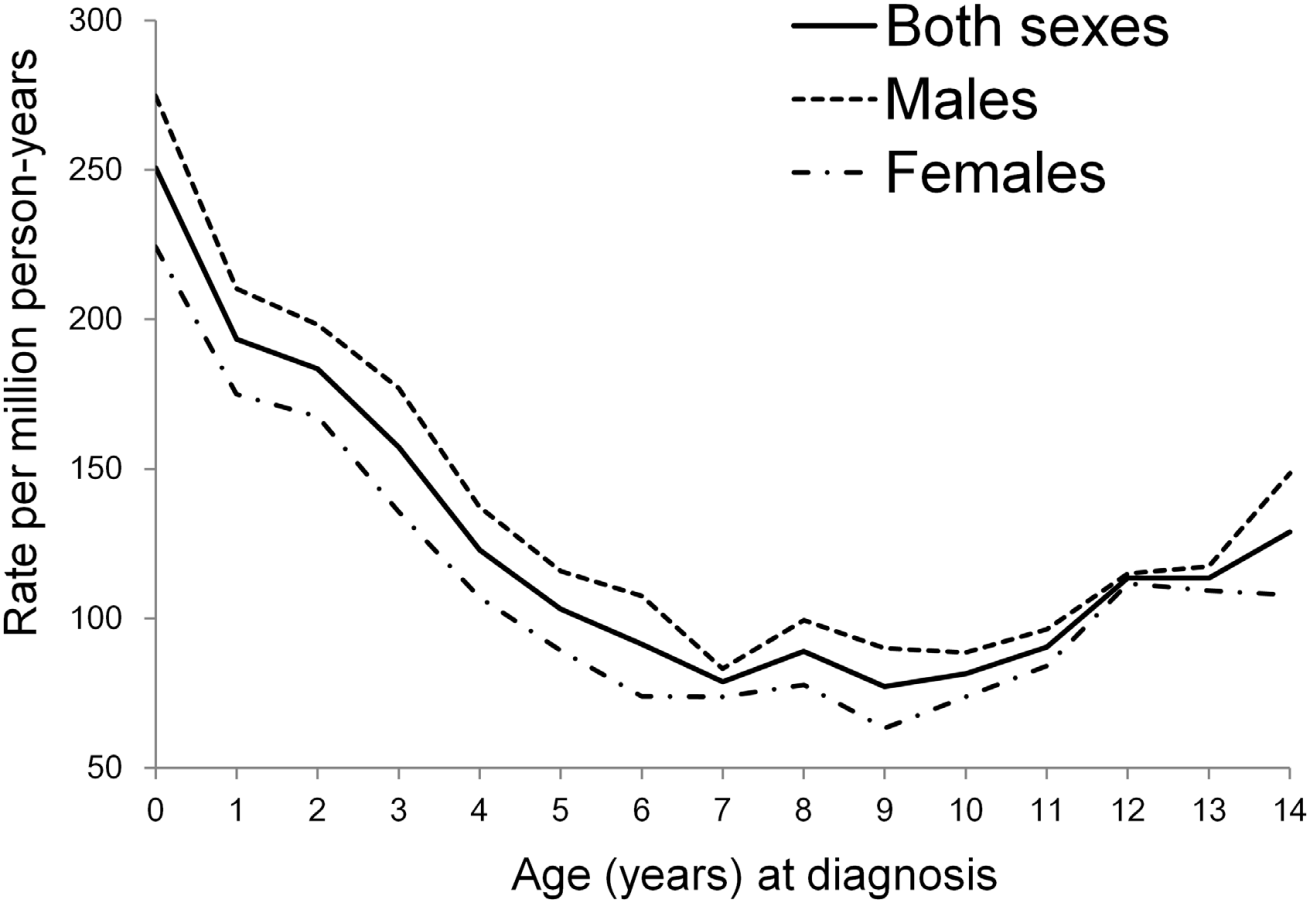
Gender-specific cancer incidence rates for children aged 0–14 years by single year of age: Taiwan, 1995–2009.

Table 1 summarizes differences in cancer incidence by sex for all infants for the years 1995 to 2009 in Taiwan. A total of 900 infants were diagnosed with cancers, giving an average of 60 cases annually. The overall incidence rate was 250.71 per million person-years. The infants represented 11% (900/8193) of all childhood cancers (aged 0–14 years) (Fig 1). Overall, leukemias (56.27 per million person-years) were the most common cancer type, followed by germ cell neoplasms (43.18 per million) and neuroblastomas (41.79 per million). The three largest groups accounted for more than half (56%) of all cancers among infants. In terms of data quality indicators defined by IARC, the DCO% for all infant cancers combined fell from 6.8% in 1995 to 0% in 2009. The MV% for all cancers combined during the 15-year study period was 95.7%. In terms of MV% according to cancer type, it varied from 86.8% (retinoblastoma) to 100% (both soft tissue sarcomas and malignant bone tumors) for most of the main groups of ICCC-3, except group XII (other and unspecified neoplasms) with a MV% of only 75% (Table 2).

**Table 1:** Gender-specific annual cancer incidence rates per million infants, by ICCC-3 group and subgroup: Taiwan, 1995 to 2009.

	Both sexes		Male		Female		M/F
ICCC-3 group	No.	Rate^a^	No.	Rate^a^	No.	Rate^a^	IRR
I Leukemias	202	56.27	100	53.36	102	59.45	0.90
Lymphoid leukemias	89	24.79	40	21.34	49	28.56	0.75
Acute myeloid leukemias	75	20.89	41	21.88	34	19.82	1.10
Chronic myeloproliferative diseases	5	1.39	4	2.13	1	0.58	-
Myelodysplastic syndrome and other myeloproliferative diseases	14	3.90	8	4.27	6	3.50	1.22
Unspecified and other specified leukemias	19	5.29	7	3.74	12	6.99	0.53
II Lymphomas	45	12.54	29	15.47	16	9.32	1.66
Hodgkin lymphomas	0	0	0	0	0	0	-
Non-Hodgkin lymphomas except Burkitt lymphoma	8	2.23	7	3.74	1	0.58	-
Burkitt lymphoma	2	0.56	2	1.07	0	0.00	-
Miscellaneous lymphoreticular neoplasms	33	9.19	18	9.60	15	8.74	1.10
Unspecified lymphomas	2	0.56	2	1.07	0	0.00	-
III CNS neoplasms	79	22.01	51	27.21	28	16.32	1.67
Ependymomas and choroid plexus tumor	16	4.46	9	4.80	7	4.08	1.18
Astrocytomas	23	6.41	14	7.47	9	5.25	1.42
Intracranial and intraspinal embryonal tumors	27	7.52	19	10.14	8	4.66	2.17
Other gliomas	7	1.95	5	2.67	2	1.17	-
Other specified intracranial and intraspinal neoplasms	1	0.28	1	0.53	0	0	-
Unspecified intracranial and intraspinal neoplasms	5	1.39	3	1.60	2	1.17	-
IV Neuroblastomas	150	41.79	88	46.96	62	36.13	1.30
Neuroblastoma and ganglioneuroblastoma	148	41.23	87	46.42	61	35.55	1.31
Other peripheral nervous cell tumors	2	0.56	1	0.53	1	0.58	-
V Retinoblastoma	68	18.94	38	20.28	30	17.48	1.16
VI Renal tumors	40	11.14	21	11.21	19	11.07	1.01
Nephroblastoma and other nonepithelial renal tumors	40	11.14	21	11.21	19	11.07	1.01
Renal carcinomas	0	0	0	0	0	0	-
Unspecified malignant renal tumors	0	0	0	0	0	0	-
VII Hepatic tumors	76	21.17	46	24.55	30	17.48	1.40
Hepatoblastoma	66	18.38	41	21.88	25	14.57	1.50
Hepatic carcinomas	3	0.84	2	1.07	1	0.58	-
Unspecified malignant hepatic tumors	7	1.95	3	1.60	4	2.33	-
VIII Malignant bone tumors	3	0.84	1	0.53	2	1.17	-
Osteosarcomas	0	0	0	0	0	0	-
Chondrosarcomas	0	0	0	0	0	0	-
Ewing tumor and related sarcomas of bone	2	0.56	1	0.53	1	0.58	-
Other specified malignant bone tumors	1	0.28	0	0.00	1	0.58	-
Unspecified malignant bone tumors	0	0	0	0	0	0	-
IX Soft tissue sarcomas	60	16.71	33	17.61	27	15.74	1.12
Rhabdomyosarcomas	20	5.57	12	6.40	8	4.66	1.37
Fibrosarcomas, peripheral nerve sheath tumors, and other fibrous neoplasms	14	3.90	7	3.74	7	4.08	0.92
Kaposi sarcoma	0	0	0	0	0	0	-
Other specified soft tissue sarcomas	20	5.57	11	5.87	9	5.25	1.12
Unspecified soft tissue sarcomas	6	1.67	3	1.60	3	1.75	-
X Germ cell neoplasms	155	43.18	95	50.69	60	34.97	1.45
Intracranial and intraspinal germ cell tumors	7	1.95	4	2.13	3	1.75	-
Malignant extracranial and extragonadal germ cell tumors	79	22.01	23	12.27	56	32.64	0.38
Malignant gonadal germ cell tumors	69	19.22	68	36.28	1	0.58	62.26
Gonadal carcinomas	0	0	0	0	0	0	-
Other and unspecified malignant gonadal tumors	0	0	0	0	0	0	-
XI Other epithelial neoplasms	18	5.01	10	5.34	8	4.66	1.14
Adrenocortical carcinomas	0	0	0	0	0	0	-
Thyroid carcinomas	1	0.28	1	0.53	0	0	-
Nasopharyngeal carcinomas	1	0.28	1	0.53	0	0	-
Malignant melanomas	4	1.11	1	0.53	3	1.75	-
Skin carcinomas	2	0.56	0	0	2	1.17	-
Other and unspecified carcinomas	10	2.79	7	0	3	1.75	2.14
XII Other and unspecified malignant neoplasms	4	1.11	3	1.60	1	0.58	-
Other specified malignant tumors	0	0	0	0	0	0	-
Other unspecified malignant tumors	4	1.11	3	1.60	1	0.58	-
Total	900	250.71	515	274.80	385	224.38	1.22

Abbreviations: CNS, Central Nervous system; ICCC-3, International Classification of Childhood Cancer version 3; M/F IRR, male-to-female incidence rate ratio.

Data include malignant tumors only.

^a^Rates were per million person-years.

-Indicates that there were fewer than 10 cases, and the statistic is not displayed in order to avoid presenting unstable data.

**Table 2:** Annual percent change for cancers among infants according to gender and ICCC-3 group: Taiwan, 1995 to 2009.

	Both sexes				Male			Female		
	Years	APC^a^	(95% CI)	MV%	Years	APC^a^	(95% CI)	Years	APC^a^	(95% CI)
ICCC-3 Group										
I Leukemias	1995–2009	2.9	(-0.9, 6.8)	97.5	1995–2009	6.9*	(1.8, 12.2)	1995–2009	-0.4	(-4.9, 4.3)
II Lymphomas		-		97.8		-			-	
III CNS neoplasms	1995–2009	5.1*	(0.3, 10.2)	93.7		-		1995–2009	6.6	(-0.7, 14.4)
IV Neuroblastomas		-		96.0		-			-	
V Retinoblastoma	1995–2009	1.3	(-3.5, 6.4)	86.8		-			-	
VI Renal tumors		-		97.5		-			-	
VII Hepatic tumors	1995–2009	8.3*	(1.7, 15.4)	89.5		-			-	
VIII Malignant bone tumors		-		100		-			-	
IX Soft tissue sarcomas	1995–2009	0.7	(-5.5, 7.2)	100	1995–2009	0.7	(-6.1, 8.1)		-	
X Germ cell neoplasms	1995–2009	1.9	(-2.2, 6.1)	98.7	1995–2009	0.1	(-5.5, 6.0)	1995–2009	5.8	(-0.3, 12.2)
XI Other epithelial neoplasms		-		94.4		-			-	
XII Other and unspecified neoplasms		-		75.0		-			-	
Total	1995–2009	2.5*	(0, 5.2)	95.7	1995–2009	3.5*	(0.6, 6.4)	1995–2009	1.3	(-1.8, 4.6)

Abbreviations: APC, annual percent change; CI, confidence interval; CNS, Central Nervous system; ICCC-3, International Classification of Childhood Cancer version 3; MV%, percentage of microscopically verified cases.

^a^The APC was calculated via weighted least-squares regression.

*Indicates statistical significance at the 0.05 level.

-Calculation of the APC was precluded by at least 1 annual rate of zero.

### Incidence rate by sex

The rates of all cancers were 274.80 and 224.38 per million person-years for males and females, respectively, giving a male-to-female incidence rate ratio (M/F IRR) of 1.22 (Table 1). Males had higher rates than females for most ICCC main groups except for leukemias. Male predominance was more pronounced (M/F IRR>1.5) for lymphomas (1.66) and CNS neoplasms (1.67).

Comparing the incidence rates according to ICCC subgroups, the subgroups with an M/F IRR greater than 2 included intracranial and intraspinal embryonal tumors (2.17) and other and unspecified carcinomas (2.14). Malignant gonadal GCTs occurred almost exclusively in males. Females had a higher incidence (M/F IRR<1) of malignant extracranial and extragonadal GCTs, unspecified and other specified leukemias, and lymphoid leukemias, with M/F IRRs ranging from 0.38 to 0.75.

### Temporal trends

**Fig 2:**
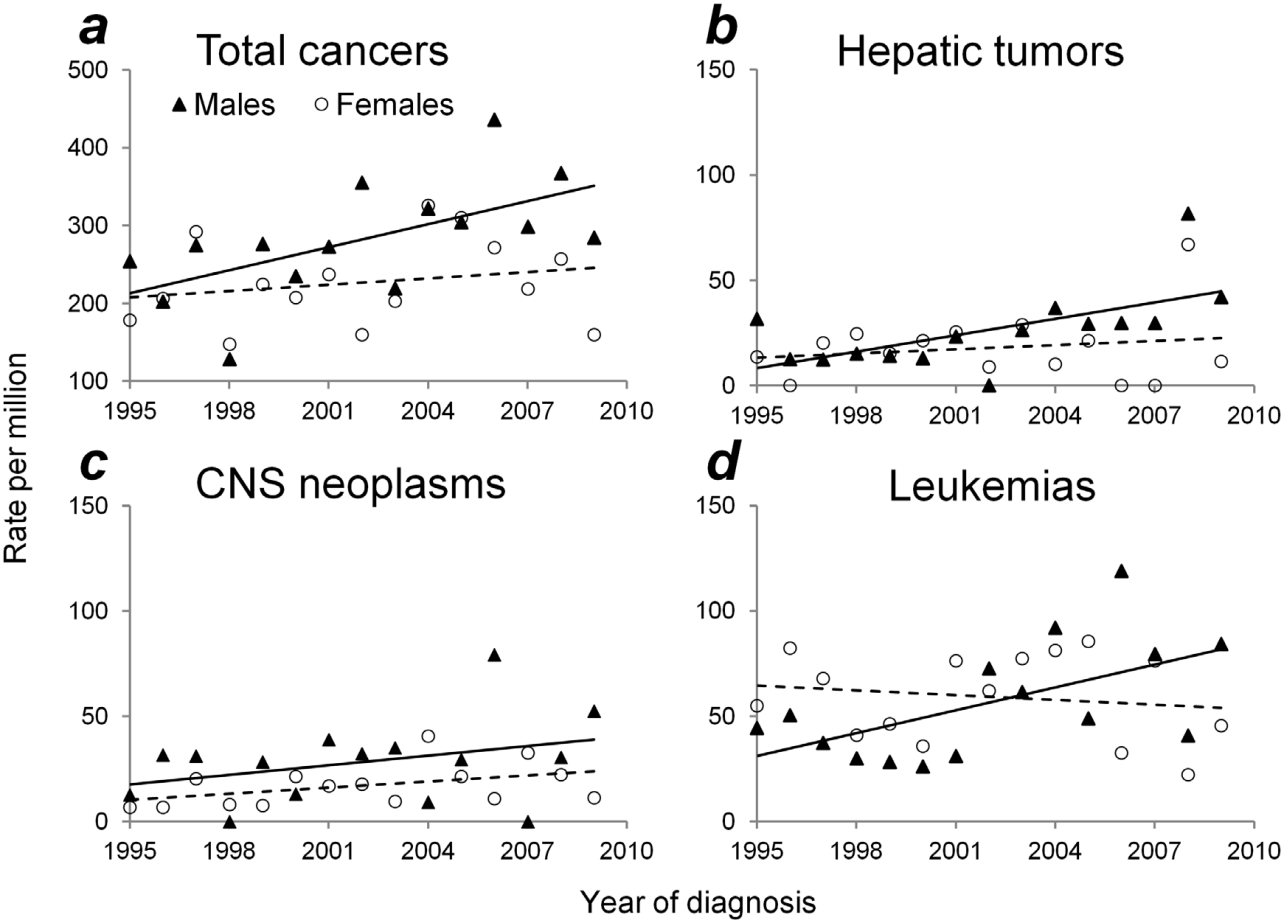
Temporal trends in cancer incidence rates among infants according to sex: Taiwan, 1995–2009. (a) Total cancers, (b) Hepatic tumors, (c) CNS neoplasms, and (d) Leukemias. Solid and broken lines: trend line for males and females, respectively.

Trends in incidence rates varied by ICCC groups (Table 2, Fig 2). Overall, the incidence rate increased 2.5% annually for all cancers combined during 1995–2009, and males had the more significant increase (APC: 3.5%, Fig 2a) than females. The overall incidence rates rose significantly for hepatic tumors and CNS neoplasms (APC: 8.3% and 5.1%; Fig 2b and 2c).

The rate of leukemias increased significantly in males during 1995–2009 (APC: 6.9%, Fig 2d), whereas no significant change in incidence trend was found among all ICCC groups for females during the study period.

### Incidence rates by country

To compare with other countries, results from the US, France, the North of England, Japan (Osaka), and China (Beijing) were compiled in Table 3 [8,10,17–19]. The incidence rate for all cancers combined in Taiwan was similar to that from the US, France and Japan (Osaka), but higher than the North of England and China (Beijing). The rates of leukemias, hepatic tumors, germ cell neoplasms, and other epithelial neoplasms were higher in Taiwan. For leukemias, the rate was as high as that in the US, Japan (Osaka) and China (Beijing), and 1.6 to 1.9 times as high as France and the North of England. In Taiwan, the rate for germ cell neoplasms was approximately 2 to 4 times greater, the rate of hepatic tumors (hepatoblastoma) was second to China (Beijing) and 2 to 5 times greater compared with the US, France, the North of England and Japan (Osaka), and the rate of “other epithelial neoplasms” was 1.4 to 2 times of that in France, North of England and Japan (Osaka). By contrast, Taiwanese infants had the lowest incidence rate for renal tumors compared with all the other countries. The rate of retinoblastoma was approximately 50% lower than the US, France, North of England and Japan (Osaka), but similar to that in China (Beijing).

**Table 3:** Comparison of infant cancer incidence rates in different countries according to ICCC-3 group.

					Rate per million (%)									
Country	USA/SEER,		France,		North of England,		Osaka Japan,				Beijing China,		Taiwan	
Years	2006–2010^a^		2000–2004^a^		1968–1995^a^		1973–87^a^ ^,^ ^c^				2000–2009^a^		1995–2009^b^	
Sex	Both		Both		Both		M		F		Both		Both	
Total no. of patients	NA		918		189		NA		NA		103		900	
ICCC-3 group														
I Leukemias	54.7	(21.5)	34.6	(14.3)	28.3	(16.9)	50	(17.0)	49.3	(19.9)	54	(26.9)	56.3	(22.4)
II Lymphomas	5.8	(2.3)	5.8	(2.4)	1.8	(1.1)	13.8	(4.7)	15.7	(6.3)	3.7	(1.8)	12.5	(5.0)
III CNS neoplasms	48.3	(18.9)	35.7	(14.7)	37.2	(22.2)	57.4	(19.6)	35.9	(14.5)	18.6	(9.3)	22.0	(8.8)
IV Neuroblastomas	49.9	(19.6)	75.1	(30.9)	31	(18.5)	33	(11.2)	37	(14.9)	24.2	(12.0)	41.8	(16.7)
V Retinoblastoma	28.4	(11.1)	29.1	(12.0)	27.5	(16.4)	26.6	(9.1)	31.4	(12.7)	16.8	(8.3)	18.9	(7.6)
VI Renal tumors	14.7	(5.8)	20.4	(8.4)	13.3	(7.9)	33	(11.2)	12.3	(5.0)	16.8	(8.3)	11.1	(4.4)
VII Hepatic tumors	9.8	(3.8)	4.2	(1.7)	4.4	(2.6)	9.6	(3.3)	12.3	(5.0)	26.1	(13.0)	21.2	(8.4)
VIII Malignant bone tumors	-	-	0.5	(0.2)	0	(0.0)	2.1	(0.7)	1.1	(0.4)	1.9	(0.9)	0.8	(0.3)
IX Soft tissue sarcomas	19.2	(7.5)	13	(5.4)	8	(4.8)	22.3	(7.6)	25.8	(10.4)	16.8	(8.3)	16.7	(6.7)
X Germ cell neoplasms	20.1	(7.9)	20.9	(8.6)	12.4	(7.4)	36.2	(12.3)	13.5	(5.5)	9.3	(4.6)	43.2	(17.2)
XI Other epithelial neoplasms	-	-	3.2	(1.3)	3.5	(2.1)	2.1	(0.7)	2.2	(0.9)	0	(0.0)	5.0	(2.0)
XII Other and unspecified malignant neoplasms	-	-	0.3	(0.1)	0	(0.0)	7.4	(2.5)	11.2	(4.5)	13.0	(6.5)	1.1	(0.4)
Total	255.0	(98.4)	242.8	(100.0)	167.4	(100.0)	293.5	(100.0)	247.7	(100.0)	201.1	(100.0)	250.7	(100.0)

Abbreviations: Both, both sexes; M, male; F, female; CNS, Central Nervous system ICCC-3, International Classification of Childhood Cancer version 3; NA, data not available; SEER, Surveillance, Epidemiology and End Results Program.

^a^Data include benign brain/CNS tumors.

^b^Data include malignant tumors only.

^c^Mass screening for neuroblastoma initiated in 1984 [20].

-Statistic could not be calculated. Rate based on less than 16 cases for the time interval [17].

Japan was the only country in which mass screening for neuroblastoma had been adopted as a national policy between 1984 and March 2004 [20]. Considering a higher incidence of neuroblastoma was likely to occur during the period, the incidence data from Japan in Table 3 was therefore confined to pre-mass screening era (1973–1987) for comparisons between countries. Furthermore, the incidence rates of CNS neoplasms were not compared because our data from the TCR in which only patients with malignant tumors were enrolled; however, both of benign and malignant CNS tumors were included in the data from the other countries.

## Discussion

Based on the nationwide population-based data of 900 cases from TCR over a period of 15 years, this study provides comprehensive cancer incidence rates and trends according to ICCC for infants in Taiwan. Comparisons between Taiwan and the other countries demonstrated significant variations in infant cancer incidence rates, with the most striking differences for hepatic tumors (hepatoblastoma) and germ cell neoplasms (Table 3).

Unlike malignant hepatic tumors in adults, in which the predominant histology is hepatocellular carcinoma, hepatoblastoma (HB) was the most common subtype in infants and accounted for 86.8% of total hepatic tumors in the current study (Table 1). As a consequence of high rate, HB also contributed to an increasing trend for hepatic tumors (APC, 8.3%, Table 2). Surprisingly, the rate of HB in Taiwan was 2 to 5 times greater compared with other countries except China (Beijing) [8]. Since the majority of population in Taiwan and China (Beijing) are Han Chinese, this novel finding of striking high rate of HB in both areas strongly suggests that genetic variations may be considered as a risk factor for HB in Han Chinese. Although the etiology is as yet unknown, recent studies have confirmed the association between HB with Beckwith-Wiedemann syndrome (BWS), familial adenomatosis polyposis (FAP), and prematurity [1,21–23]. BWS and FAP were two of the well-known cancer predisposition syndromes associated with childhood cancers [24–26]. The evaluation of the risk of HB in BWS and FAP revealed a relative risk of 2,280 and 1,220 during the first four years of life [25,26]. The association between HB and prematurity or very-low-birth-weight (VLBW) was first shown in Japan and confirmed in other studies [22,23], suggesting the possibility of factors associated with prematurity and its treatment may play a role in the occurrence of HB. In Taiwan, the association between these well-known risk factors and HB remain undetermined. Nevertheless, similar to the findings in industrialized countries, advances in obstetrical and neonatal care also have led to an increase in the survival of VLBW infants in Taiwan [27], which may account for the notable upward trend for HB seen in this analysis.

The etiologies for germ cell neoplasms in infants and adolescents were distinct. Besides, the genetic alterations and histologic subtypes varied between these two age groups [1]. Therefore, the influence of age should be taken into consideration while comparing the rates with different countries. Evidence suggested that there were racial variations in subtypes of childhood GCTs [1,28]. However, studies of this disease entity on infants are scarce. Based on the data in the present study, the overall incidence rate of infantile germ cell neoplasms in Taiwan was approximately 2 to 4 times greater compared with other countries in Western World (including the US, France and North of England) and Eastern Asia (Osaka, Japan and Beijing, China) (Table 3). Our observation provided strong evidence on racial variations between Taiwanese (Han Chinese) and White (Caucasian) infants. The unique expression pattern of miRNAs in Taiwanese pediatric CNS GCTs shown in a previous study suggest that genomic differences are likely to play an important role in the observed racial variations [29]. However, further genetic studies to elucidate the differences in other subgroups of infant GCTs and comparison with other races are still warranted.

Our analysis has shown that cancer incidence among infants in Taiwan significantly increased by 2.5% annually from 1995 to 2009. This overall increasing trend in cancer incidence was predominantly contributed by male infants (Table 2); however, the factors responsible for this observation are unclear. The 6.8% decline of DCO% over the study period suggest that the possibility of improvements in ascertainment of all data sources resulted in the overall increase cannot be completely excluded. Although such improvements may play a role, our findings could not be fairly interpreted by this reasoning. First, only certain cancer groups had upward incidence trends. Second, the average increase was only found among males but not females. These findings contradicted the hypothesis for the improvements mentioned above that were expected to proportionately affect all cancer type-specific and sex-specific patient groups. In addition, the homogeneously high quality in cancer diagnosis throughout the 15-year study period, as evidenced by the small numbers of cases in group XII of ICCC (0.4%) over the study period, provided no evidence supporting a role of cancer registration improvement in the overall increase. On the basis of these findings, we concluded that the improvement in cancer registration only marginally influenced the incidence trends.

There were other limitations in this study. The change in morphology classification and diagnostic technology over time might cause misclassification biases on these estimates. In addition, biased comparisons between countries could be introduced as a consequence of multiple factors. Because the rarity of infant cancers, small numbers of patients in some subtypes were more likely to fluctuate yearly and led to unstable statistics. Also, data from the other countries comprised both benign and malignant brain/CNS tumors [8,10,17–19], Taiwan had a lowered rate in this context (ICCC III and Xa) could be a consequence of the TCR included malignant CNS tumors only. These informations should be borne in mind when interpreting the variations between countries and temporal changes in the cancer incidence rates specifically for infants.

## Conclusions

Our analysis suggests that cancer incidence among infants increased over time in Taiwan, and the increase was specific for males. However, the factors responsible for our findings are unclear. An increased incidence of hepatoblastoma was noted, and was likely to be associated with increased survival in prematurity. Racial variations in cancer incidence among infants were demonstrated by comparing with other countries. The incidence rates of hepatoblastoma and germ cell neoplasms in Taiwanese infants to be 2–4 times that seen in Western countries were the most striking differences. Further investigations may be performed in other areas of similar ethnicity to explore the potential impacts of environmental factors on carcinogenesis of these cancers while controlling for race.
